# Posterior Transpedicular Dynamic Systems in the Treatment of Chronic Lumbar Instability

**DOI:** 10.1155/2013/432520

**Published:** 2013-08-07

**Authors:** Ali Fahir Ozer, Vijay K. Goel, Ahmet Alanay, Mehdi Sasani, Tunc Oktenoglu, Deniz Erbulut

**Affiliations:** ^1^Department of Neurosurgery, Koc University Medical School, Sariyer, 34450 Istanbul, Turkey; ^2^Engineering Center for Orthopaedic Research Excellence (E-CORE), Departments of Bioengineering and Orthopaedic Surgery, Colleges of Engineering and Medicine, University of Toledo, Toledo, OH 43606, USA; ^3^Departments of Orthopaedics and Traumatology, Bilim University, Faculty of Medicine, Istanbul Spine Center, Florence Nightingale Hospital, Sisli, 34403 Istanbul, Turkey; ^4^Department of Neurosurgery, American Hospital, Nisantasi, 34365 Istanbul, Turkey; ^5^Departments of Mechanical Engineering and Neurosurgery, Koc University, Sariyer, 34450 Istanbul, Turkey

Dynamic stabilization is a new concept and a new technology in spinal stabilization. There are a lot of debates on this topic and most of the criticisms may be true; however, it should be kept in mind that every technology develops from necessity. If fusion could solve all the problems, scientists would not try to develop other solutions. 

Posterior dynamic stabilization is a hopeful technology and we believe that tremendous innovative surgical techniques and surgical instruments will develop in near future. We expect that we will open new horizons in your minds when you are considering treatment plans about your patients which need lumbar stabilization procedures. In this special issue, you will find several examples of dynamic stabilization surgeries for different spinal diseases. Clinical results were also given with expanded review of the relevant literature.

The papers in this journal also open a new window about treatment of lumbar disc herniations. Type 2 and type 4 disc herniations, according to the Carragee classification, treated with dynamic stabilization instead of subtotal discectomy were discussed by A. F. Ozer et al. 

You will find manuscripts in this special issue regarding recurrent disc herniations, painful black discs, degenerative spondylolisthesis, and painful Modic degenerations with degenerative disc disease treated with posterior dynamic stabilization surgery. We believe that dynamic systems will be discussed more intensely in the treatment of degenerative disc and bony pathologies in the lumbar spine.

There are a lot of biomechanical and clinical studies about dynamic stabilization systems of the lumbar spine. Recently, finite element studies also have been published frequently concerning these systems. D. U. Erbulut et al. have performed an excellent review on biomechanics of posterior dynamic stabilization systems, and readers will learn too much from this paper in this special issue.

We know that load sharing of the spinal column is an important biomechanical factor which may directly affect a patient's pain level, fusion success, and future disease progression, following spine surgery. In this journal, there is also a well-written research about load-sharing and regional load distribution of the interbody area investigating comparisons with between rigid rods, semirigid PEEK rods, and semirigid dynamic posterior instrumentation with flexion-extension dampening materials.

There is a very important paper about adult's degenerative scoliosis which concluded that pedicle screw-based dynamic stabilization can be used in elderly patients with mild degenerative lumbar scoliosis because this is a less invasive surgery with short operative duration, moderate blood loss, and low adverse event rates. Moreover, they also reported scoliosis curve stabilization, at an average followup of more than 5 years. 

It was believed that fusion is a gold standard for low back pain treatment so far. However, there have been several complications reported clinically. These complications mainly are related to pseudoarthrosis and adjacent segment degeneration due to high stiffness at the stabilized segment. As an alternative treatment, nonfusion stabilization systems became more and more popular in order to preserve mobility of a motion segment and eliminate adjacent segment phenomena. You will see research studies emphasizing the benefits of dynamic stabilization system to prevent pseudoarthrosis and adjacent segment disease in this issue. 

The posterior transpedicular dynamic stabilization method is a very good surgical procedure in the patients with segmental instability. When we consider the biomechanical problems including widening of neutral zone and weakness of parts which stabilize the spine, one can easily understand the effectiveness of dynamic systems in treating patients who have lumbar segmental instability. 

There are also reports about the benefits of interspinous spacers and the results in terms of pain control, motion preservation, and prevention of adjacent segment degeneration. The authors in this journal recommend its use in treatment as well as in prevention of adjacent segment disease specifically in young patients where spinal fusion for early degenerative disease is needed.

We know that dynamic stabilization of the lumbar spine not only stops the degeneration process but also starts the regeneration. L.-Y. Fay et al. reported the rehydration of intervertebral disc after dynamic system surgery using MRI evaluation. These papers prove radiological improvement of the disc tissue after this surgery. 

*Ali Fahir Ozer* *Vijay K. Goel* *Ahmet Alanay* *Mehdi Sasani* *Tunc Oktenoglu* *Deniz Erbulut*

